# Effects of quercetin on *in vitro* rumen fermentation parameters, gas production and microflora of beef cattle

**DOI:** 10.3389/fmicb.2025.1527405

**Published:** 2025-04-30

**Authors:** Ming Xiao, Liu Du, Manlin Wei, Yajing Wang, Chenyang Dong, Ji Ju, Runze Zhang, Wen Peng, Yuxiang Wang, Yongjie Zheng, Weijing Meng

**Affiliations:** ^1^College of Animal Science and Technology, Inner Mongolia Minzu University, Tongliao, China; ^2^College of Animal Science and Technology, China Agricultural University, Beijing, China; ^3^Tongliao Agriculture and Animal Husbandry Development Centre, Tongliao, China

**Keywords:** quercetin, beef cattle, *in vitro*, rumen fermentation, gas production, microflora

## Abstract

Methane is an important component of greenhouse gases, and ruminant production is a significant source of methane emissions. At present, flavonoid feed additives have certain applications in methane inhibition in ruminants. However, the effects of different doses of quercetin on rumen fermentation parameters, rumen bacteria and archaea are still unclear. Therefore, this study investigated the effects of quercetin on *in vitro* rumen fermentation parameters, methane production, and microflora in beef cattle. A completely randomized design was adopted. Quercetin was added to the fermentation substrates at 0% (group C), 0.5% (group Q1), 1% (group Q2) and 1.5% (group Q3). Anaerobic fermentation was carried out at 39°C for 48 h, gas production (GP) was recorded at different times, gas composition was determined, and methane (CH_4_) production was calculated. Fermentation parameters and dry matter digestibility (DMD) were determined after 48 h. Moreover, rumen fluid was collected for rumen bacterial and archaeal flora determination. The results were as follows: (1) After 32 h of fermentation, the GP decreased in response to the addition of quercetin. With increasing quercetin concentration, the theoretical maximum gas production decreased quadratically before 20 h (*P*_quadratic_ = 0.032). There was a quadratic increase in gas production (*P*_quadratic_ = 0.024). With increasing quercetin supplementation, the NH3-N content increased quadratically (*P*_quadratic_ = 0.027). MCP increased linearly and quadratically with quercetin (*P*_linear_ = 0.002, *P*_quadratic_ = 0.005), whereas DMD decreased linearly and quadratically with quercetin (*P*_linear_ = 0.013, *P*_quadratic_ = 0.032). Both 0.5 and 1% quercetin significantly reduced the butyrate content (*P*_quadratic_ = 0.002). With the addition of quercetin, the levels of butyrate, isobutyrate, isovalerate, and total volatile fatty acid (TVFA) first decreased but then increased (*P*_quadratic_ < 0.05). (2) With increasing quercetin concentration, methane production (*P*_quadratic_ = 0.009) and the methane proportion (*P*_quadratic_ < 0.001) decreased quadratically. (3) The ACE index and Chao1 index increased quadratically with quercetin supplementation (*P*_quadratic_ < 0.05). The relative abundance of *Succiniclasticum* in groups Q1 and Q3 increased, whereas the relative abundances of *norank_f__norank_o__Rickettsiales* and *Curtobacterium* decreased in all quercetin groups at the genus level (*P* < 0.05). (4) Quercetin supplementation did not affect the diversity of the archaeal community, but the relative abundance of *Methanobrevibacter* in group Q2 decreased. Overall, quercetin influenced *in vitro* rumen fermentation and the bacterial flora to decrease methane production and promote rumen nitrogen utilization and MCP synthesis.

## 1 Introduction

With the increasing impact of global climate change on ecosystems and human society, reducing greenhouse gas emissions, especially carbon dioxide and methane emissions, has become the focus of the international community. In animal husbandry, ruminants are considered one of the major sources of greenhouse gas emissions, with rumen methane production accounting for 80% of the total gastrointestinal methane production (Liebig et al., 2010). Methane emissions from ruminants are estimated to account for approximately 16% of global greenhouse gas emissions, with cattle and sheep accounting for approximately 70% and 20%, respectively (Lan and Yang, 2019). Methane in the rumen is produced mainly by the reduction of carbon dioxide by hydrogen, which is then excreted by ruminant hiccups (Soroye et al., 2020). In addition, the large amount of methane produced by rumen fermentation causes 2%−15% of feed energy to be converted to methane and lost (Ellis et al., 2007). Therefore, the methane produced by rumen fermentation in ruminants not only aggravates the greenhouse effect but also leads to the loss of feed energy. Therefore, inhibiting rumen methane production not only helps improve feed energy utilization efficiency but also effectively reduces the greenhouse effect.

Currently, various measures have been taken to reduce CH_4_ emissions from ruminants. These interventions include improving farm systems, optimizing forage, implementing dietary interventions, and adding methanobacteria inhibitors and feed additives (Belanche et al., 2020). The addition of certain plant extracts to diets has been proven to be simple and effective. Studies have shown that flavonoids have a direct effect on methanogens (Patra and Saxena, 2010; Bodas et al., 2012; Purba et al., 2019; Sinz et al., 2018). Kim et al. (2015) also reported that flavonoid-rich plant extracts can reduce methane emissions. In addition, Oskoueian et al. (2013) reported that flavonoids can inhibit the formation of methane. These findings have potential application value as rumen regulators for CH_4_ reduction in ruminants.

Quercetin is a flavonoid that is widely present in nature (Nam et al., 2016), is found in a variety of plants and forages, and has good anti-inflammatory (Carullo et al., 2017), antibacterial (Kim et al., 2022), antioxidant (Lespade, 2016), and anticancer effects (Geng et al., 2019). Flavonoids have been shown to improve the production performance of ruminants and maintain rumen health by reducing the bald area of the rumen wall and maintaining the pH, which could reduce the incidence of rumen acidosis (Bodas et al., 2012; Balcells et al., 2012). In addition, flavonoids, quercetin, and quercetin-containing plants reduce CH_4_ production in the rumen (Purba et al., 2019; Sinz et al., 2018). However, the effects of different doses of quercetin on rumen fermentation parameters, rumen bacteria and archaea are still unclear. Therefore, the purpose of this study was to evaluate the effects of quercetin on rumen fermentation, CH_4_ production and microflora in beef cattle via an *in vitro* method to provide a reference for the application of quercetin in ruminant rumen regulation and animal production.

## 2 Materials and methods

### 2.1 Materials

The quercetin used in this experiment was provided by Xi'an Musen Bioengineering Co., Ltd., Shanxi Province, China, with a purity of ≥98%, and the rumen fluid used in this experiment was collected from three Simmental crossbred bulls (bodyweights of 550 kg ± 30 kg) provided by the experimental base of Tongliao Mufeng Jiuyuan Husbandry Co., Ltd.

### 2.2 Composition and nutrient levels of the fermentation substrate

The substrate used for fermentation was the same as that used in the diet of the rumen fluid donor cattle, which was prepared according to the “Nutritional Requirements for Beef Cattle” (NASEM., 2016). The feed composition and nutrient levels of the substrate are shown in Table 1.

**Table 1:** The composition and nutrient levels of the substrate (DM basis).

**Ingredients**	**Content, %**	**Nutrient levels^b^**	**Content, %**
Corn Straw	40.00	Metabolizable energy (MJ/kg)	10.08
Corn	33.00	Dry matter	90.26
Distillers Dried Grains with Solubles	2.00	Ether extract	2.30
Soybean meal	14.00	Crude protein	12.62
Corn germ meal	6.00	Neutral detergent fiber	39.70
Limestone	1.80	Acid detergent fiber	25.26
Calcium hydrogen phosphate	1.20	Calcium	1.24
Salt	1.00	Phosphorus	0.56
Premix^a^	1.00		
Total	100.00		

^a^The guaranteed level of premix (per kg) is as follows: 100 mg of Fe, 100 mg of Zn, 30 mg of Cu, 40 mg of Mn, 6,000 IU of vitamin A, 40 IU of vitamin E, and 2,000 IU of vitamin D_3_. ^b^The metabolizable energy was calculated on the basis of nutrient values provided by NASEM. (2016), whereas the other values were measured.

### 2.3 Experimental design

In this study, a completely randomized design was adopted. According to the dry matter calculation, quercetin was added to the fermentation substrates at 0% (group C), 0.5% (group Q1), 1% (group Q2), and 1.5% (group Q3), with 3 replicates in each group. The *in vitro* gas production (GP), methane proportion, dry matter digestibility (DMD), and fermentation parameters, including pH value, ammonia nitrogen (NH_3_-N), and volatile fatty acids (VFAs), were determined. Furthermore, the bacterial and archaeal flora of the rumen fluid were also analyzed.

### 2.4 Determination methods

#### 2.4.1 Collection and treatment of rumen fluid

Two h before the morning feeding, the rumen fluid of 3 Simmental cattle was drawn through the esophagus via a vacuum sampler. To avoid the influence of saliva, the first 200 mL of rumen fluid from each cattle was discarded, and approximately 200 mL of rumen fluid was drawn and evenly mixed before being filtered through four layers of gauze and quickly transferred into a dispenser to form the artificial rumen culture fluid (rumen fluid: buffer = 1:2) according to the methods of Menke and Steingass (1988). The buffer in the dispenser was preheated (39°C) and purged to maintain anaerobic conditions with CO_2_.

#### 2.4.2 Determination of *in vitro* gas production and methane production

The substrate of approximately 0.2000 g dry matter (DM) was weighed into a 100 mL graduated glass syringe (model HFT000025, Häberle Company, Germany), with 0% (control), 0.5%, 1%, or 1.5% quercetin added, and the glass syringe was preheated to 39°C. Approximately 30 mL of artificial rumen culture mixture was added to a 100 mL graduated glass syringe preheated at 39°C. The syringes were kept in a constant-temperature water bath shaker at 120 r/min at 39°C for 48 h. GP in each glass syringe was recorded at 0, 1, 2, 4, 6, 8, 12, 16, 20, 24, 32, 40, and 48 h, and the gas was collected in a 200 mL aluminum foil bag (Shanghai Huibin Instrument Co., Ltd., Shanghai, China).

The theoretical maximum gas production and the gas production rate were estimated according to the model of Ørskov and McDonald (1979):


$$\begin{array}{l} Y=a+B\times \left(1-e^{-ct}\right) \end{array}$$

where Y, a, B, c, and t represent the gas production (mL), the gas production from the immediately soluble fraction (mL), the theoretical maximum gas production (mL), the gas production rate (mL/h), and the incubation time (h), respectively.

The proportions of hydrogen (H_2_), CH_4_ and CO_2_ in the gas sampling bag (%, v/v) were determined via a gas chromatograph (model TP-2060T, Beijing Analytical Instrument Co., Ltd.). The instrument conditions were as follows: TCD detector, column model TDX-01, 1 m × 3 mm × 2 mm, column temperature of 120°C, detector temperature of 150°C, injection port temperature of 150°C, argon carrier gas flow rate of 50 mL/min, standard gas concentration of 24.80% CH_4_, 65.10% CO_2_, 2.01% H_2_, 3.00% O_2_, and 5.00% N_2_, and an injection volume of 0.1 mL. Methane production was calculated from total gas production and the proportion of methane produced at 48 h of fermentation.

#### 2.4.3 Determination of *in vitro* dry matter digestibility

The dry matter digestibility (DMD) was measured according to the two-stage method of Tilley and Terry (1963): approximately 0.3000 g of substrate was weighed and put into a special bag (Beijing-Beef and Cattle Information Technology Research Center, size 35 mm × 75 mm, pore size 38–40 μm), with 3 replicates in each group. Three bags were placed in a 100 mL culture tube, in which 35 mL of artificial rumen fluid (rumen fluid: buffer = 1:1) was added, and the tubes were incubated anaerobically at 39°C for 48 h. After that, the bags were removed and rinsed with distilled water to remove the rumen fluid, and 35 mL of pepsin solution was added to the culture tube to perform another 48 h of anaerobic culture at 39°C. The bag was subsequently removed, rinsed with distilled water to remove pepsin, and dried at 105°C to a constant weight to calculate the DMD according to the following equation:


$$\begin{array}{l} DMD=\frac{m-\left(m_{2}-m_{1}\times c\right)}{m}\times 100\% \end{array}$$

where DMD is the *in vitro* dry matter digestibility (%); m is the DM weight of the sample (g); m_1_ is the DM weight of the empty bag (g); m_2_ is the DM weight of the bag and the sample residue (g); and c is the correction factor of the blank bag (the DM weight after incubation/the DM weight before incubation).

#### 2.4.4 Determination of rumen fermentation parameters

The syringes were immediately placed in ice water to terminate fermentation after 48 h. Part of the fermentation residue was transferred into a 5 mL centrifuge tube to determine the microbial protein (MCP) content according to the method of Sedmak and Grossberg (1977). The other fermentation residue was transferred into a 50 mL centrifuge tube to determine the pH value immediately with a portable pH meter (Testo 205, Germany), followed by centrifugation (8,000 r/min, 4°C for 15 min). The supernatant was collected for the determination of NH_3_-N and VFAs according to the colorimetric methods of Verdouw et al. (1978) and Chen et al. (2022). The gas chromatographic parameters (model GC-6800, Beijing Beifen Tianpu Instrument Technology Co., Ltd.) were as follows: Φ6 mm × 2 m quartz glass-packed column, column temperature of 150°C, injection port temperature of 220°C, injection volume of 1 μL, and FID detector temperature of 280°C. The carrier gas was high-purity N_2_, the flow rate was 30 mL/min, and the pressure was 200 kPa. The gas used was H_2_, and the flow rate was 30 mL/min. The auxiliary gas used was air, and the flow rate was 300 mL/min.

#### 2.4.5 Determination of bacteria in the rumen

Five milliliters of the fermentation residue in the glass syringe was transferred to a cryopreservation tube and stored at −80°C for further determination. The tube was placed on a sterile platform and thawed in a mixture of ice water. Total DNA extraction was performed according to the E.Z.N.A.^®^ soil kit (Omega Biotek, Norcross, GA, U.S.). A NanoDrop 2000 was used to determine the DNA concentration and purity, and 1% agarose gel electrophoresis was used to determine the quality of the extracted DNA. PCR amplification was performed via bacterial V3-V4 region primers 338F (5′-ACTCCTACGGGAGGCAGCAG-3′) and 806R (5′-GGACTACHVGGGTWTCTAAT-3′), and the resulting PCR products were purified, washed, and detected after recovery. The Illumina MiSeq PE300 platform was used for sequencing (Shanghai MegiBiomedical Technology Co., Ltd.).

#### 2.4.6 Determination of archaea in the rumen

The V4-V5 variable region of the 16S rRNA gene was amplified via PCR via 524F10ext (5′-TGYCAGCCGCCGCGGTAA-3′) and Arch958RmodR (5′-YCCGGCGTTGAVTCCAATT-3′) after DNA extraction. There were 3 PCR repeats per sample, and the products were detected after mixing 3 repeats of PCR products, recovered products, purified and detected, and quantified with a Quantus™ fluorometer (Promega, USA). The purified PCR products were constructed via the NEXTFLEX Rapid DNA-Seq Kit and sequenced via Illumina's MiSeq PE300/NovaSeq PE250 platform (Shanghai Magi Biomedical Technology) Limited Company.

### 2.5 Data statistics and analysis

The gas production at each time point and the rumen fermentation parameters at 48 h were collated with Microsoft Excel 2010 (Microsoft Corp., USA). Duncan's method in SPSS 22 (SPSS Inc., USA) was used for analysis of variance and multiple comparisons. Moreover, orthogonal polynomial contrasts were used to analyse the linear and quadratic effects of different doses of quercetin. The rumen microflora data were analyzed for differences via the Kruskal–Wallis test. The results were considered significant at *P* < 0.05, and the results are expressed as the means and means of standard errors (SEMs). All microbial data were analyzed via the biological cloud platform (https://cloud.majorbio.com). UPARSE software (http://drive5.com/uparse/, version 7.1) was used to cluster the sequences according to 97% similarity. Taxonomic annotation of OTU species was performed by comparing the 16S rRNA gene database (Silva v138) with the RDP classifier, and the community composition of each sample was calculated at different species classification levels. In addition, partial least square discriminant analysis (PLS-DA) was used. Mothur software was used to calculate and draw dilution curves and Venn diagrams according to the OTU information. LEfSe analysis (LDA > 3.5) was used to identify the bacterial groups with significant differences in abundance from phylum to genus. The Spearman correlations between the rumen microorganisms and the rumen fermentation parameters were subsequently analyzed.

## 3 Results

### 3.1 Effect of quercetin on *in vitro* gas production

**Figure 1:**
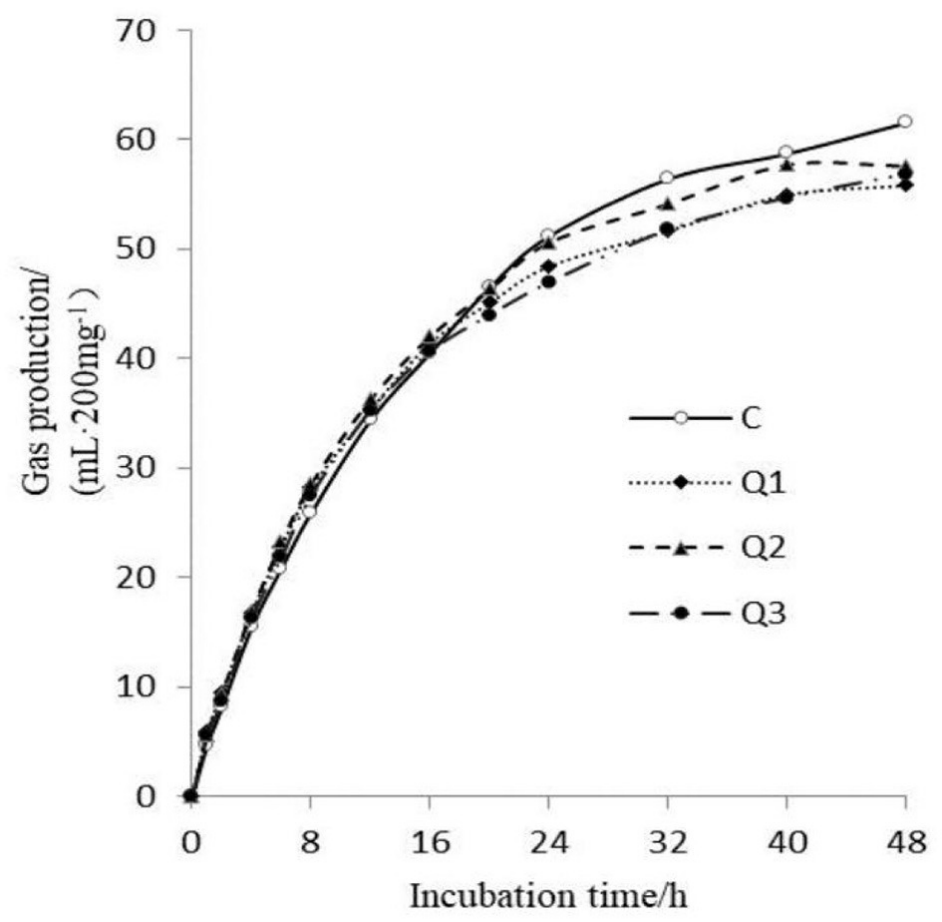
Gas production at different times during 48 h of *in vitro* rumen fermentation.

As shown in Figure 1, quercetin had no effect on GP at the initial stage of *in vitro* fermentation, but after 32 h of fermentation, quercetin reduced GP. As shown in Table 2, with increasing quercetin supplementation level, the theoretical maximum gas production decreased quadratically before 20 h (*P*_quadratic_ = 0.032). Gas production showed a secondary increase (*P*_quadratic_ = 0.024), but the increase was small between the treatments. With increasing quercetin concentration, methane production (*P*_quadratic_ = 0.009) and the methane proportion (*P*_quadratic_ < 0.001) decreased quadratically. Compared with those in the control group, when 1.5% quercetin was added, the methane production and methane proportion were reduced by 15.82% and 8.78%, respectively.

**Table 2:** Effect of quercetin on *in vitro* rumen gas production.

**Items**	**Groups**				**SEM**	***P*** **value**	
	**C (0%)**	**Q1 (0.5%)**	**Q2 (1%)**	**Q3 (1.5%)**		**Linear**	**Quadratic**
a (mL)	0.50	1.03	0.82	0.97	0.389	0.475	0.692
B (mL)	63.97^a^	55.69^b^	58.41^b^	56.14^b^	1.236	0.030	0.027
c (h^−1^)	0.06^b^	0.09^a^	0.08^a^	0.08^a^	0.004	0.118	0.006
Methane production (mL)	12.07^a^	10.91^b^	11.07^b^	10.16^b^	0.264	0.002	0.009
Methane proportion (%)	19.60^a^	19.53^a^	19.22^a^	17.88^b^	0.184	< 0.001	< 0.001

Groups C, Q1, Q2, and Q3 represent 0%, 0.5%, 1% and 1.5% quercetin addition, respectively; ^a^gas production from the immediately soluble fraction (mL); ^b^theoretical maximum gas production; ^c^gas production rate. In the same row, values with different superscript letters are significantly different (*P* < 0.05), whereas those with the same letter or no superscript letter are not significantly different (*P* > 0.05).

### 3.2 Effect of quercetin on rumen fermentation parameters and dry matter digestibility

As shown in Table 3, there was no statistically significant difference in pH among all the groups (*P* > 0.05), and the values were within the normal range (6.68–6.72). However, NH_3_-N increased quadratically (*P*_quadratic_ = 0.027) with increasing quercetin concentration. MCP increased linearly and quadratically (*P*_linear_ = 0.002, *P*_quadratic_ = 0.005), whereas DMD decreased linearly and quadratically with increasing quercetin concentration (*P*_linear_ = 0.013, *P*_quadratic_ = 0.032). After 48 h of *in vitro* culture, the addition of 0.5% quercetin significantly reduced butyrate and isovalerate (*P*_quadratic_ = 0.002 and 0.019, respectively), and 1% quercetin significantly reduced butyrate, isobutyrate, isovalerate, and total volatile fatty acid (TVFA) (*P*_quadratic_ = 0.002, 0.043, 0.019 and 0.027, respectively), whereas 1.5% quercetin significantly increased propionate (*P*_linear_ = 0.052) but had no significant effect on the other VFAs.

**Table 3:** Fermentation parameters and dry matter digestibility after 48 h of *in vitro* rumen fermentation.

**Items**	**Groups**				**SEM**	***P*** **value**	
	**C (0%)**	**Q1 (0.5%)**	**Q2 (1%)**	**Q3 (1.5%)**		**Linear**	**Quadratic**
pH value	6.68	6.72	6.70	6.70	0.017	0.288	0.536
NH_3_-N (mg/dL)	19.68^b^	20.16^b^	20.92^a^	20.26^ab^	0.215	0.083	0.027
MCP (mg/mL)	44.00^b^	48.19^ab^	50.18^a^	51.64^a^	1.364	0.002	0.005
Acetate (mmol/L)	29.85	28.57	27.63	29.95	0.556	0.863	0.034
Propionate (mmol/L)	6.13^b^	6.97^ab^	6.36^b^	7.44^a^	0.282	0.052	0.160
Butyrate (mmol/L)	6.54^a^	5.82^b^	5.06^c^	6.52^a^	0.166	0.684	0.002
Isobutyrate (mmol/L)	0.81^a^	0.75^a^	0.61^b^	0.79^a^	0.031	0.453	0.043
Valerate (mmol/L)	0.58^ab^	0.53^a^	0.56^b^	0.66^b^	0.030	0.109	0.028
Isovalerate (mmol/L)	0.45^a^	0.33^bc^	0.31^c^	0.42^ab^	0.035	0.705	0.019
A/P	4.93	4.10	4.36	4.02	0.241	0.060	0.121
TVFA (mmol/L)	44.34^ab^	42.93^bc^	40.53^c^	45.78^a^	0.827	0.767	0.027
DMD (%)	61.73^a^	60.68^b^	61.00^b^	60.85^b^	0.175	0.013	0.032

A/P represents the ratio of acetic acid to propionic acid; TVFA represents total volatile fatty acid; DMD represents dry matter digestibility. In the same row, values with different superscript letters are significantly different (*P* < 0.05), whereas those with the same letter or no superscript letter are not significantly different (*P* > 0.05).

### 3.3 Effect of quercetin on rumen bacteria

#### 3.3.1 Changes in bacterial diversity in the rumen

**Figure 2:**
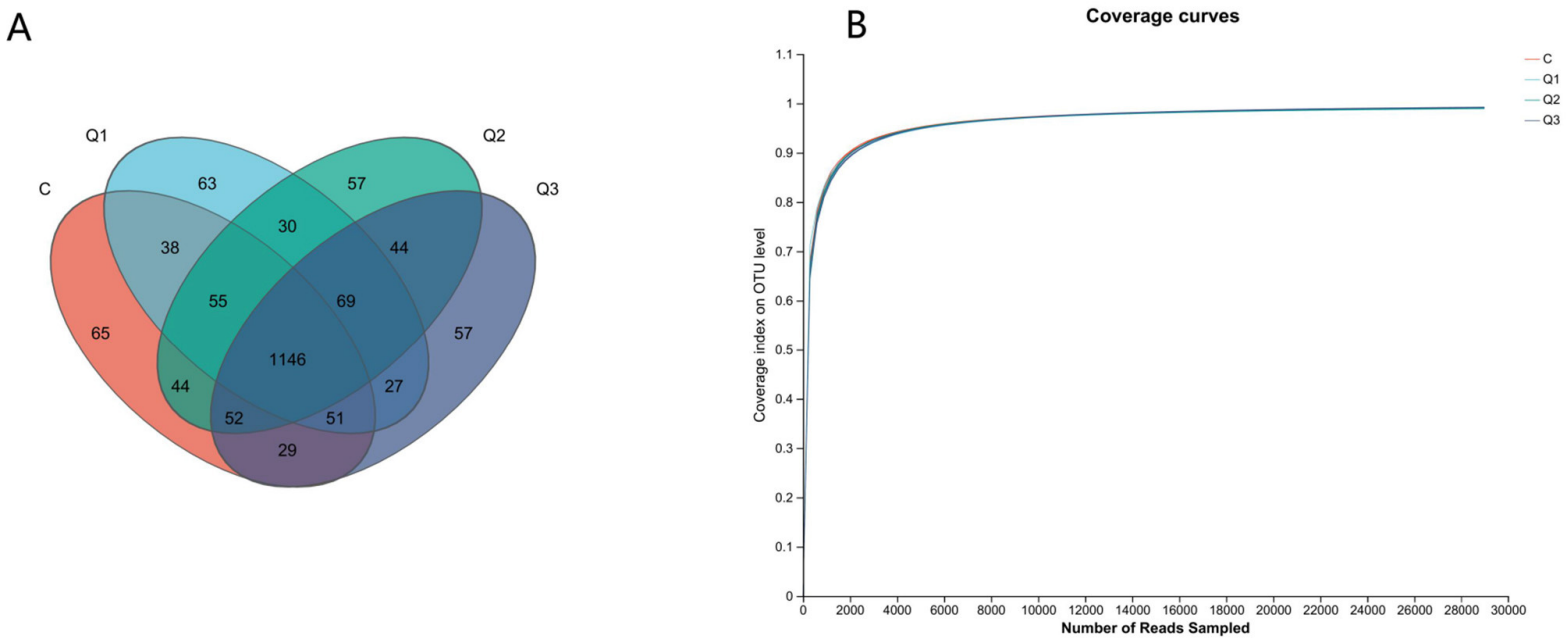
**(A)** Operational taxonomic unit-Venn (OTU-Venn) analysis; **(B)** dilution curve analysis.

UPARSE software was used to cluster each sample at the 97% similarity level, and a total of 1,827 OTUs were obtained (Figure 2A). As shown by the coverage curve (Figure 2B), the rumen microbial coverage of both the quercetin group and the control group exceeded 99%, indicating a high sequencing depth, which could indicate the rationality of the sequencing quantity and depth of the sequencing samples in this study. As shown in Table 4, the Ace index and Chao index increased quadratically with quercetin supplementation (*P* < 0.05), whereas the other indices were not affected by quercetin.

**Table 4:** Effects of quercetin on the rumen bacterial diversity index of beef cattle.

**Items**	**Groups**				**SEM**	***P*** **value**	
	**C (0%)**	**Q1 (0.5%)**	**Q2 (1%)**	**Q3 (1.5%)**		**Linear**	**Quadratic**
Coverage(%)	99.995	99.999	99.997	99.997	1.102	0.802	0.253
Ace index	1,311.50	1,412.20	1,385.30	1,314.90	25.790	0.914	0.009
Chao index	1,308.22^b^	1,443.54^a^	1,368.20^ab^	1,316.23^b^	24.456	0.776	0.021
Shannon index	5.19	5.01	5.30	5.24	0.165	0.518	0.716
Simpson index	0.02	0.04	0.02	0.02	0.012	0.626	0.693

In the same row, values with the different superscript letters are significantly different (*P* < 0.05), whereas those with the same letter or no superscript letter are not significantly different (*P* > 0.05).

#### 3.3.2 Effect of quercetin on bacterial composition in the rumen

**Figure 3:**
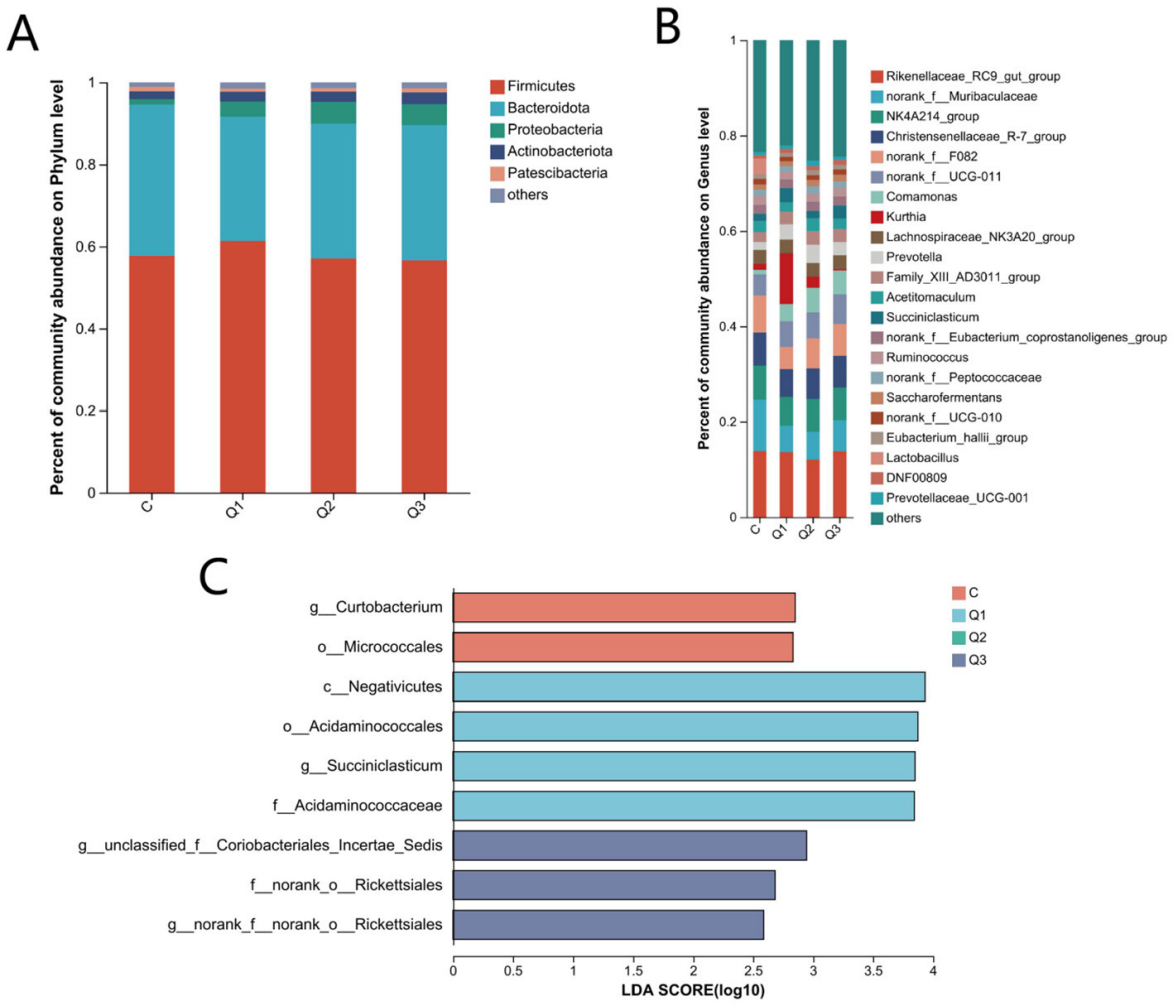
**(A)** Relative abundance of rumen bacteria at the phylum level; **(B)** relative abundance of rumen bacteria at the genus level; **(C)** significantly different bacterial taxa identified by linear discriminant analysis effect size (LEfSe).

At the taxonomic level, a total of 21 phyla, 41 classes, 96 orders, 162 families, 314 genera, 575 species, and 1,827 OTUs were obtained. At the phylum level, *Firmicutes, Bacteroidetes, Proteobacteria, Actinobacteria*, and *Patescibacteria* were the dominant phyla. The relative abundance was >1%, and the relative abundances of *Bacteroideaceae* and *Firmicutes* were the highest among all the groups, accounting for more than 88% of the total abundance (Figure 3A). At the phylum level, there was no significant difference among the groups (*P* > 0.05). At the genus level, there were 17 genera with relative abundances >1.0%, of which *Rikenellaceae_RC9_gut_group, NK4A214*, and *unranked norank_f_Muribaculaceae* were the dominant bacterial genera in each group (Figure 3B). Compared with those in group C, the relative abundances of *Succiniclasticum* in groups Q1 and Q3 were significantly greater (*P* < 0.05), whereas those of *norank_f__norank_o__Rickettsiales* and *Curtobacterium* were significantly lower in the quercetin groups (*P* < 0.05). The LEfSe results (Figure 3C) revealed significant differences in 2, 4, and 3 biomarkers in groups C, Q1 and Q3, respectively (LDA score > 3.5).

#### 3.3.3 Correlation analysis of the rumen bacterial composition, the rumen fermentation parameters and methane production

**Figure 4:**
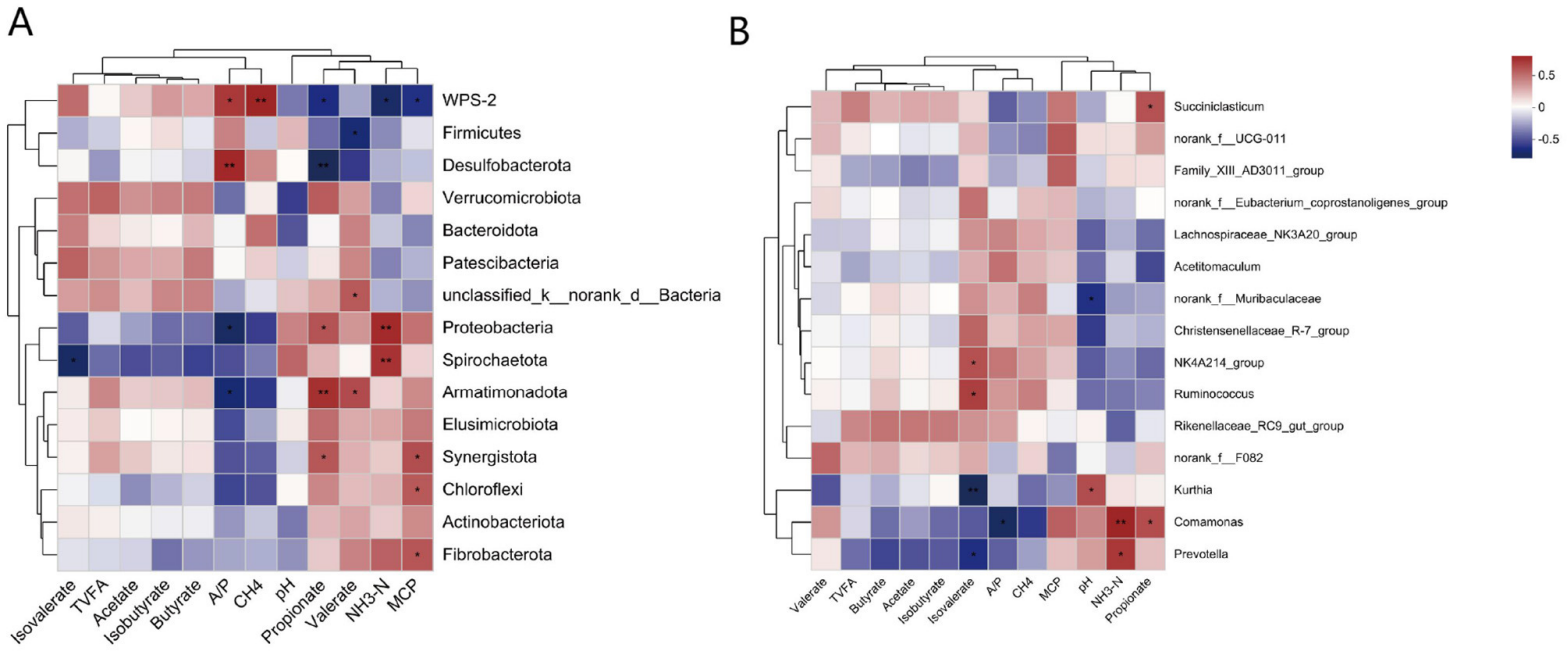
**(A)** Pearson correlation heatmap analysis of the bacterial phyla with rumen fermentation parameters and methane production. **(B)** Pearson correlation heatmap analysis of the bacterial genuses with rumen fermentation parameters and methane production. *, *P* < 0.05; **, *P* < 0.01; ***, *P* < 0.001. The legend in the upper right corner is the color interval of the different correlation R values.

The correlation analysis results are shown in Figure 4. WPS-2 (*P* < 0.05) and *Desulfobacterota* (*P* < 0.01) were significantly and positively correlated with A/P. *Proteobacteria* and *Armatimonadota* were significantly negatively correlated with A/P (*P* < 0.05). WPS-2 was positively correlated with CH_4_ (*P* < 0.01). There was a significant positive correlation between propionate and *Proteobacteria* (*P* < 0.05), *Armatimonadota* (*P* < 0.01), and *Synergistota* (*P* < 0.05). *Desulfobacterota* was significantly negatively correlated with propionate (*P* < 0.05) and WPS-2 (*P* < 0.05). *Unclassified_k_norank_d_Bacteria* and *Armatimonadota* were significantly positively correlated (*P* < 0.05), whereas *Fibrobacterota* and valerate were significantly negatively correlated. *Proteobacteria* (*P* < 0.01) and Synergistota (*P* < 0.01) were significantly positively correlated with NH_3_-N, whereas WPS-2 was significantly negatively correlated with NH_3_-N. *Spirochaetota* (*P* < 0.05), *Chloroflexi* (*P* < 0.05), and *Fibrobacterota* (*P* < 0.05) were significantly positively correlated with MCP, whereas WPS-2 was significantly negatively correlated with MCP (*P* < 0.05).

*Succiniclasticum* was significantly positively correlated with propionate, whereas *norank_f_Muribaculaceae* was significantly negatively correlated with pH. *NK4A214_group* and *Ruminococcus* were significantly positively correlated with isobutyrate, whereas *Kurthia* was significantly negatively correlated with isobutyrate. *Comamonas* was positively correlated with propionate, negatively correlated with A/P and positively correlated with NH_3_-N. *Prevotella* was significantly negatively correlated with isobutyrate and positively correlated with NH_3_-N.

### 3.4 Effect of quercetin on rumen archaea

#### 3.4.1 Changes in archaeal diversity in the rumen

**Figure 5:**
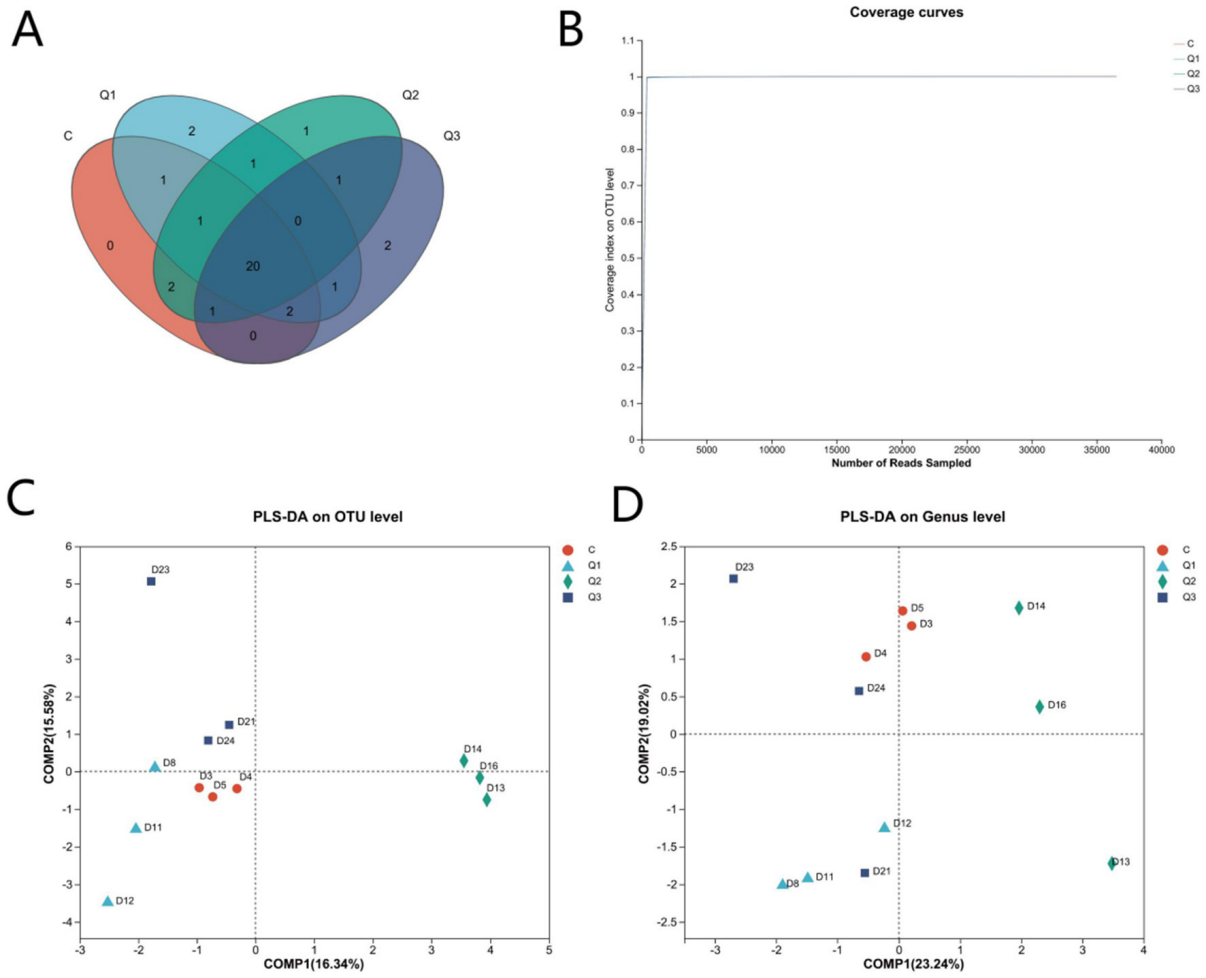
**(A)** Operational taxonomic unit-Venn (OTU-Venn) analysis; **(B)** dilution curve analysis; **(C)** PLS-DA analysis of quercetin on rumen archaea at the OUT level; **(D)** PLS-DA analysis of quercetin on rumen archaea at the genus level.

Each sample was clustered and labeled at a 97% similarity level via UPARSE software (Figures 5A, B). As shown in Figures 5C, D, the contribution rate of the first principal component was 16.34%, and the contribution rate of the second principal component was 15.58% at the OTU level. At the genus level, the contribution rate of the first principal component was 23.24%, and the contribution rate of the second principal component was 19.02%, which could be clearly distinguished among groups Q1, Q2, and C, indicating that the compositions of the control group and the treatment groups in the rumen fluid samples were significantly different. Table 5 shows that quercetin had no significant effect on the richness or diversity of the rumen archaeal communities (*P* > 0.05).

**Table 5:** Effects of quercetin on the rumen archaeal diversity index of beef cattle.

**Items**	**Groups**				**SEM**	***P*** **value**	
	**C (0%)**	**Q1 (0.5%)**	**Q2 (1%)**	**Q3 (1.5%)**		**Linear**	**Quadratic**
Coverage(%)	99.995	99.999	99.997	99.997	0.001	0.077	0.200
Ace index	36.47	22.59	27.90	26.14	6.193	0.090	0.140
Chao index	29.00	21.50	23.58	26.21	4.467	0.383	0.478
Shannon index	1.04	1.02	1.03	1.01	0.014	0.483	0.780
Simpson index	0.47	0.48	0.49	0.50	0.008	0.534	0.830

In the same row, values with different superscript letters are significantly different (*P* < 0.05), whereas those with the same letter or no superscript letter are not significantly different (*P* > 0.05).

#### 3.4.2 Effect of quercetin on the archaeal community in the rumen

**Figure 6:**
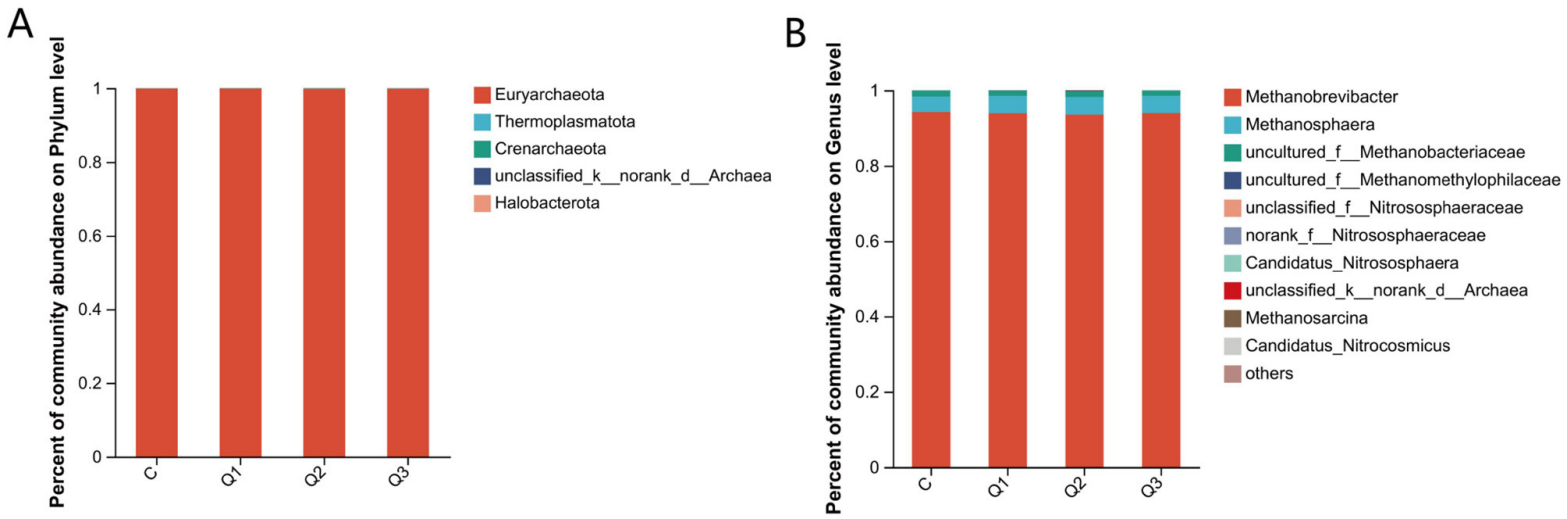
**(A)** Relative abundance of rumen archaea at the phylum level; **(B)** relative abundance of rumen archaea at the genus level.

The relative abundance distribution of the archaeal community at the phylum level is shown in Figure 6A. There was no significant difference between the groups (*P* < 0.05), and more than 99% of the archaea in each group were *Euryarchaeota*.

The relative abundance at the genus level is shown in Figure 6B. The archaea with relative abundances higher than 1.0% included *Methanobrevibacter, Methanosphaeria*, and unclassified *Methanobacteriaceae*. All groups were dominated by *Brevibacterium methanescens*, but the content of each group differed. The content of *Brevibacterium methanescens* in group Q2 (93.56%) was significantly lower than that in group C (94.19%) and group Q1 (93.87%) (*P* < 0.05), and the content in group Q3 was in the middle (93.96%).

#### 3.4.3 Correlation analysis of the rumen archaeal composition with fermentation parameters and methane production

**Figure 7:**
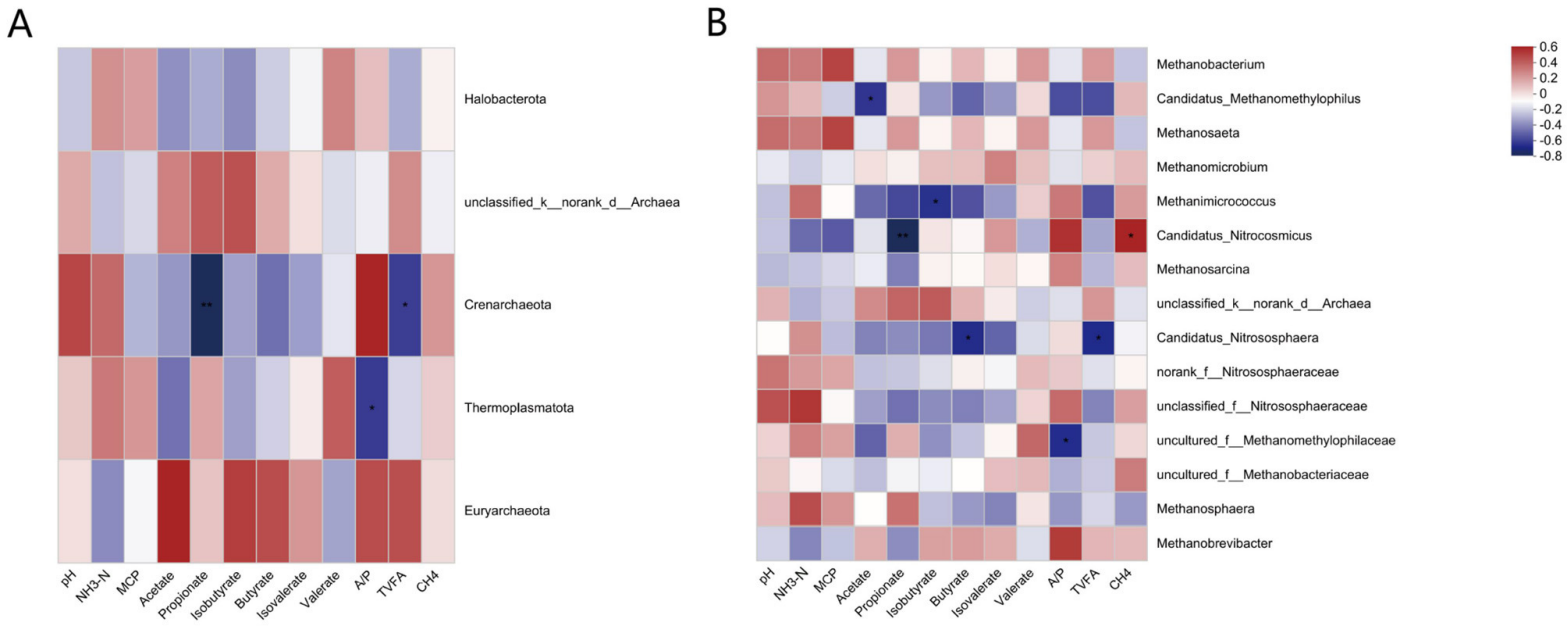
**(A)** Pearson correlation heatmap analysis of the archaeal phyla with rumen fermentation parameters and methane production. **(B)** Pearson correlation heatmap analysis of the archaeal genus with rumen fermentation parameters and methane production. *, *P* < 0.05, **, *P* < 0.01, ***, *P* < 0.001. The legend in the upper right corner is the color interval of the different correlation R values.

The results of the correlation analysis between the rumen archaeal composition and fermentation parameters and methane production at the phylum level are shown in Figure 7A. *Crenarchaeota* was significantly negatively correlated with propionate and negatively correlated with TVFAs. *Thermoplasmatota* was negatively correlated with A/P.

The results of the correlation analysis between the rumen archaeal composition and VFA and methane production are shown in Figure 7B. The *Candidatus Methanomethylophilus* was significantly negatively correlated with acetate. The abundances of *Methanicrococcus* and *isobutyrate* were significantly negatively correlated. *Candidatus Nitroscosmicus* was significantly negatively correlated with propionate and significantly positively correlated with CH_4_ production. *Candidatus_Nitrososphaera* was significantly negatively correlated with butyrate and TVFAs. *uncultured_f_Methanomethylophilaceae* was also negatively correlated with A/P.

## 4 Discussion

### 4.1 Effect of quercetin on *in vitro* rumen gas production

Gas production reflects the degradation of dry matter, especially carbohydrates, in feed (Liu et al., 2002). Therefore, *in vitro* gas production can be used as a reference index to measure feed degradability. Moreover, it can also preliminarily reflect the number of fermentable substances in the rumen, the activity of microorganisms and the availability of substrates (Patra and Saxena, 2010). Generally, an increase in the fermentability of nutrients in the rumen or an increase in microbial activity will increase the rumen GP. Flavonoids have different effects on rumen GP (Wang et al., 2022). Some studies have shown that flavonoids reduce total rumen GP (Amin et al., 2021). However, other studies have shown that quercetin and some flavonoid extracts increase rumen GP (Leiber et al., 2012). On the one hand, the effect of flavonoids on the rumen GP is related to the antibacterial effect of the flavonoids themselves (Cushnie and Lamb, 2005). In addition, the composition of the fermentation substrate and the rumen environment also influence the rumen GP. In this study, the decrease in the rumen GP after 32 h of fermentation and the theoretical maximum gas production may be related to the decreased DMD of the substrate, which is also reflected in the VFA results. Moreover, according to the microflora results, rumen bacteria such as *Proteobacteria, Synergistota, Armatimonadota*, and *Proteobacteria* were identified. WPS-2 and *Desulfobacterota, Candidatus_Nitrocosmicus, Candidatus_Nitrososphaera, Methanimicrococcus*, and *Candidatus Methanomethylophilus* may also influence the results. On the other hand, the gas production rate increased at the early phase (before 20 h) of fermentation, which indicated that the fermentation of carbohydrates by some microorganisms was promoted, and the composition of these microorganisms remains to be further studied. Since this experiment was conducted under simulated conditions *in vitro*, which could not fully replicate the dynamic rumen environment, further *in vivo* experiments are still needed to verify the results.

Rumen microorganisms produce large amounts of CO_2_, CH_4_, H_2_, and nitrogenous gas during the degradation of dietary carbohydrates and other nutrients, and it is thought that the production of CH_4_ is related to the reduction of CO_2_ by methanogens via H_2_ in acetate and butyrate fermentation (Bata and Rahayua, 2017). In this study, quercetin reduced the CH_4_ content in rumen gas (Table 2) but also decreased the acetate and butyrate concentrations in group Q2 and decreased the abundance of *B. methane*, which further confirmed the above inference. Several *in vitro* experiments reported that quercetin reduced CH_4_ production by 43% after 48 h (Nørskov et al., 2023), and even plant leaves containing quercetin had significant CH_4_-inhibiting effects (Purba et al., 2019). This may be related to the widespread antimicrobial properties of flavonoids (Cushnie and Lamb, 2005). Since methanogens live on rumen ciliates (protozoa) and can use the hydrogen produced by the former to synthesize CH_4_ (Vogels et al., 1980), the inhibitory effect of quercetin on protozoa indirectly reduces CH_4_ production (Kim et al., 2015). This is similar to the effect of tea saponin on reducing rumen CH_4_ production (Guo et al., 2008; Hess et al., 2004), but it is different from the effect of tannins because tannins bind directly with proteins on the surface of methanogens to reduce CH_4_ (Nørskov et al., 2023). Ruminant animals can use their ingested energy more effectively by reducing methane emissions, thereby improving growth performance and production efficiency (Sun et al., 2023). Moreover, by reducing methane emissions, the sustainability of agriculture can be improved, and the burden on the environment can be reduced (Nørskov et al., 2023). Therefore, reducing methane emissions has important nutritional and ecological importance.

### 4.2 Effect of quercetin on *in vitro* rumen fermentation parameters and dry matter digestibility

pH is an important index for evaluating the acid production of rumen microorganisms and is affected by factors such as saliva secretion, rumen organic acid accumulation, and the ratio of grain to roughage. Studies have shown that the pH value of rumen fluid fluctuates between 5.5 and 7.5 and that excessively acidic and alkaline environments are not conducive to the survival of rumen microorganisms or rumen fermentation (Feng et al., 2020). Under *in vitro* experimental conditions, the rumen pH value excludes the influence of saliva entry and the secretory function of the rumen wall and is completely dependent on the acid produced by substrate fermentation. The results of this study revealed that quercetin reduces the concentration of TVFAs in the rumen and promotes the production of alkaline substances (NH_3_-N), which is conducive to maintaining the normal pH value of the rumen; these findings are consistent with those of Purba et al. (2019), who recommended 0.1–15 mg of *Piper betle* L. that contains 1.84% quercetin on a DM basis.

The NH_3_-N concentration can be affected by decomposed protein in feed by microorganisms (Zheng et al., 2021) and can also reflect the utilization of protein by rumen microorganisms. Therefore, the NH_3_-N concentration is an important indicator of the utility of protein in the rumen. Thao et al. (2014) reported that NH_3_-N concentrations ranging from 5–30 mg·dL^−1^ were most suitable for rumen microbial growth and that the higher the NH_3_-N concentration was, the more MCP was synthesized (Lu et al., 2019). MCP can meet 60%−70% of the protein requirements of ruminants and is the main nitrogen source of ruminants and an important indicator reflecting the nitrogen use efficiency of microorganisms (Zheng et al., 2020). In this study, with increasing quercetin supplementation, the NH_3_-N and MCP concentrations increased quadratically, indicating that quercetin favors the utilization of nitrogen in the rumen. On the other hand, the increase in microorganisms can also promote the degradation of nitrogenous substances in the rumen, thereby increasing the NH_3_-N concentration in the rumen (Ramos-Morales et al., 2018).

The DMD reflects the overall digestion of nutrients in the rumen and is an important indicator of the nutritional value of feed, and a high DMD is usually due to the strong activity of rumen microbes and fuller fermentation (Kamra et al., 2006). Sinz et al. (2018) showed that there was no significant difference in the effect of 4.5% quercetin on DMD compared with the control group. In this study, the addition of quercetin decreased DMD in linear and quadratic forms (P linear = 0.013, P quadratic = 0.032). According to the study of Cushnie and Lamb (2005), this result may be due to the antibacterial properties of flavonoids. It may also be that the activity of cellulolytic bacteria and amylolytic bacteria reduces DMD.

VFAs can provide approximately 70%−80% of the energy available for ruminant metabolism. Acetate is one of the main products of rumen microbial activity, the main decomposition product of cellulose and hemicellulose, and as the main precursor of ruminant fat synthesis, it is essential for the synthesis of milk fat and body fat (Jian et al., 2020). Propionate is mainly used for the synthesis of body fat and lactose and can be converted into glucose through hepatic gluconeogenesis to supply energy; the greater the proportion of propionate is, the more energy it can provide to the body (Ballard, 1972). Fermentation of propionate (usually an acetate to propionate ratio < 3) is also conducive to weight gain in beef cattle. Moreover, the production of propionate competitively consumes a large amount of H_2_, which in turn inhibits the production of CH_4_ (Janssen, 2010). In this study, a significant increase in the propionate concentration and a decrease in the CH_4_ content in the rumen gas were also observed in group Q3. This is also confirmed by the results of Kamra et al. (2006) and Patra and Saxena (2010). That is, the addition of flavonoids can reduce the production of rumen CH_4_ and reduce A/P. TVFA was generally positively correlated with DMD, and the decrease in DMD in this study led to a significant decrease in the TVFA concentration in group Q2, which was related to the significant decrease in acetate and butyrate in this group. However, there was no significant difference in TVFA concentration between groups Q1 and Q3.

### 4.3 Effect of quercetin on *in vitro* rumen bacteria

In this study, the Ace index and Chao index increased quadratically with quercetin supplementation (*P* < 0.05), indicating that the addition of quercetin may increase the richness of microorganisms but does not affect the diversity of the rumen microbiota, as demonstrated by Oskoueian et al. (2013). The results of this study are consistent with those of previous reports and are likely related to the antibacterial effects of flavonoids on certain bacterial phyla and genera.

The dominant bacterial phyla in the rumen fluid include *Firmicutes, Proteobacteria, Bacteroidetes, Actinomycetes*, and *Patellar Bacteria*, and their abundance changes with rumen digestion (Thao et al., 2014). In this study, *Firmicutes* and *Bacteroidetes*, which work together to hydrolyse and synthesize carbohydrates and proteins (Bäckhed et al., 2005), were the dominant phyla in the rumen, followed by *Proteobacteria, Actinomycetes*, and *Patellar Bacteria*. On the other hand, the sum of *Firmicutes* and *Bacteroidetes* in group C (94.55%) was greater than that in the other quercetin groups, and the lowest was in group Q3 (89.59%). This suggests the inhibitory effect of quercetin on the degradation of nutrients in the rumen, which is consistent with the reduction in the rumen GP in group Q3. This is caused mainly by the antibacterial action of quercetin, which has been reported in many studies (Li et al., 2024; Wang et al., 2018; Aljadaan et al., 2020).

At the genus level, the abundance of *Succiniclasticum* increased with the addition of quercetin. It also accounts for the increase in propionate content in groups Q1 and Q3 because of its ability to degrade starch, fiber and cellobiose as fermentation substrates and convert succinic acid to propionate (Li et al., 2018). Succinic acid can be converted to propionic acid through a variety of pathways, one of which is that Acetyle-CoA enters the citrate cycle (TCA cycle) and finally synthesizes propionic acid through the succinic acid pathway or lactic acid pathway (Mu et al., 2021). In terms of rumen gas production, methanogens are thought to be able to use metabolites (including acetate, propionate, CH_4_, CO_2_, and H_2_) produced by other microorganisms to synthesize CH_4_ with H_2_ and CO_2_, which ensures the relative stability of the partial pressure of hydrogen in the rumen (Leiber et al., 2012). However, when the abundance of *Succiniclasticum* increased in the rumen, it competed with hydrotrophic methanogens, which also explained why quercetin reduces CH_4_ production via a molecular mechanism. Although *norank_f__norank_o__ Rickettsiales* and *Curtobacterium* are also significantly reduced, they account for a very low proportion of the microflora and may therefore have a relatively low impact on CH_4_ production.

### 4.4 Effect of quercetin on *in vitro* rumen archaea

Archaea are thought to be most closely associated with methane emissions. However, there is no such correlation between methane emissions and the total abundance of archaea (Tapio et al., 2017). This happens not only to cattle but also to sheep (Kittelmann et al., 2013). In this study, there was no significant difference in the Chao1 index, ACE index, Shannon index or Simpson index between the quercetin groups and the control group, indicating that the addition of quercetin affected methane production without affecting the diversity or richness of the archaea.

Methanogens are the main archaea in the rumen (Janssen and Kirs, 2008). Approximately 89.3% of the total number of archaea in the rumen fluid are *Methanobacteridae* (Janssen and Kirs, 2008). In this study, 99% of the archaea at the phylum level were euryarchaeota, and the relative abundance of *Methanobacteridae* was similar in every group. These findings further confirmed that rumen methane production is not necessarily related to archaea. Rumen methane production is a complex process in which methanogens cooperate with bacteria, protozoa and fungi to form a symbiotic system in which CO_2_, formic acid, acetate, ethanol, and methyl compounds (methanol, monomethylamine, dimethylamine, or dimethyl sulfur) are used to carry out carbon cycling. Although most archaea, such as *B. methane* and *B. vannamei*, can use H_2_ and CO_2_ to produce CH_4_ through the hydrotrophic pathway (Sharp et al., 1998; Danielsson et al., 2017), other ways in which methane can be produced (Qiao et al., 2014). Flavonoids (including quercetin) inhibit methane production (Patra and Saxena, 2010; Tavendale et al., 2005). The same results were also observed in this study, but only *Methanobrevibacter* in group Q2 was significantly reduced, indicating that quercetin had a greater effect on gas-producing bacteria than on rumen archaea. However, whether quercetin has other influences or a specific effect at a certain dose remains to be confirmed.

## 5 Conclusions

In summary, quercetin decreased *in vitro* rumen gas production and methane production, increased the enriched NH_3_-N and MCP concentrations, and decreased the abundance of *Brevibacterium methanescens* in the rumen. Supplementation with 1.5% quercetin significantly increased the rumen propionate concentration, increased the abundance of *rumen succinate bacteria*, and decreased the relative abundances of *norank_f__norank_o__Rickettsiales* and *Curtobacterium*. Overall, quercetin influenced *in vitro* rumen fermentation and the rumen bacterial flora to decrease methane production and promote rumen nitrogen utilization and MCP synthesis.

## Data Availability

The original contributions presented in the study are publicly available. This data can be found here: https://www.ncbi.nlm.nih.gov/, accession numbers: PRJNA1141169, PRJNA1141186.
